# Assessment of cardiac function by speckle tracking echocardiography in children with osteogenesis imperfecta

**DOI:** 10.1038/s41390-025-04522-y

**Published:** 2025-11-19

**Authors:** Saeed Elseedy, Shimaa Elnemr, Shaimaa Badreldeen, Heba ElSayed Dawoud, Mona Hashim, Doaa El Amrousy

**Affiliations:** 1 Ministry of Health and Population, Tanta, Egypt; 2 Pediatric Department, Faculty of Medicine, Tanta University, Tanta, Egypt

## Abstract

**Background:**

We aimed to assess the subclinical cardiac dysfunction in children with osteogenesis imperfecta (OI) using two-dimensional speckle tracking echocardiography (2D-STE).

**Methods:**

This cross-sectional study was carried out on 30 children with OI and 30 healthy children of matched age and sex. All children underwent conventional and tissue Doppler echocardiography, as well as 2D-STE.

**Results:**

Height, weight, and hemoglobin were significantly lower in the patient group than in the control group. Aorta (AO), left atrial diameter (LAD), left ventricular end-systolic diameter (LVESD), left ventricular end-diastolic diameter (LVEDD), right ventricular diameter (RVD), and mean pulmonary artery (MPA) were significantly larger in the patient group compared to the control group. Mitral E/é, LV S, LV 2D-global longitudinal strain (2D-GLS), TAPSE, RV S, and RV 2D-GLS were significantly lower in the patient group than the control group. There was a significant positive correlation between the clinical severity of disease and (AO, LAD, LVESD, LVEDD, MPA, and RVD). There was a significant negative correlation between the severity of disease and LV 2D-GLS, RV é/á, and RV 2D-GLS.

**Conclusion:**

Children with OI have subclinical LV and RV systolic and diastolic dysfunction, and these abnormalities were correlated with the clinical severity of the disease.

**Impact:**

- Osteogenesis imperfecta (OI) is an inherited disease of abnormal collagen 1 with extra-skeletal involvement, such as cardiovascular complications.
- Aortic dilatation and valvular dysfunction have been reported in adult patients with OI. However, right and left ventricular function assessment using two-dimensional speckle tracking echocardiography has not been studied in pediatrics before.
- We found that children with OI have subclinical LV and RV systolic and diastolic dysfunction, and these abnormalities are correlated with the clinical severity of the disease. Regular echocardiographic follow-up is recommended for these patients.

## Introduction

Osteogenesis imperfecta is a rare hereditary disorder that affects connective tissue, especially collagen fibers, characterized by fragile bone and increased risk for fracture. Depending on how severe the skeletal symptoms are, there are various types of OI.^1^ Autosomal dominant inheritance of COL1A1 and COL1A2 gene mutations accounts for the majority of osteogenesis imperfecta cases. Type I collagen chains, which are responsible for the structure and strength of bone and other structures, are encoded by these genes; thus, patients with OI exhibit extra-skeletal involvement such as the cardiovascular system.^2^

Type I collagen is found in the heart’s valves, fibrous rings, tendinous cords, vascular adventitia, and interventricular septum; the mitral valve’s collagen composition is roughly 75% type I collagen. The ventricular myocardium’s collagen fibers support myocyte architecture and are responsible for its rigidity. Collagen types III and I are also abundant in the aorta and the majority of arteries.^3^ Adult patients with osteogenesis imperfecta have been reported to have myocardial dysfunction, aortic dilatation, and valve disease.^4–6^ However, studies in pediatrics are few.

This study aimed to assess the subclinical cardiac dysfunction in children with OI using two-dimensional speckle tracking echocardiography (2D-STE) to correlate it to the clinical severity of the disease.

## Patients and methods

This cross-sectional study was carried out on 30 children with Osteogenesis Imperfecta (OI) who were admitted to the genetic unit, Pediatric department, Tanta University Hospitals, and from outpatient genetics clinics from July 2023 to July 2024 after approval of the institutional ethical committee. Thirty healthy children of matched age & sex served as the control group. Informed written consent was obtained from the parents of all included children.

### Inclusion criteria

Pediatric patients aged less than 18 years of both sexes who were diagnosed with OI.

#### Exclusion criteria

Children with known congenital heart disease or acquired heart diseases such as rheumatic fever, children with systemic hypertension or previous cardiac surgery, children with arrhythmias, children with systemic diseases, and children with hepatic or renal impairment.

The enrolled children were categorized into two equal groups:

- Group I (patient group): included thirty children diagnosed with OI.
- Group II (control group): included thirty healthy children of matched age and sex.

All children underwent detailed history taking and clinical examination for age, sex, height, weight, blood pressure, age at diagnosis of OI, the time of first fracture, number of fractures, consanguinity, and history of surgery.

Routine laboratory investigations included complete blood count (CBC), renal function tests (serum creatinine and urea), and liver function tests [Total bilirubin, direct bilirubin, alanine aminotransferase (ALT), aspartate aminotransferase (AST)]. Clinical severity was assessed according to the scoring system proposed by Aglan et al.^7^ and patients were divided into mild, moderate, and severe categories.

#### Echocardiography

Echocardiography examinations were performed with an ultrasound device (model iE33, Philips Medical Systems) with an S5-1 MHz transducer by a single expert pediatric cardiologist.

### Conventional echocardiography

2D echocardiography and M-mode echocardiography: were obtained in the standard parasternal, apical, and subcostal views to measure left ventricular end diastolic diameter (LVEDD), left ventricular end systolic diameter (LVESD), aortic diameter (AO), left atrial diameter (LAD), mean pulmonary artery (MPA) diameter, and right ventricular diameter (RVD). Interventricular septum (IVS) and LV posterior wall (LVPW) were measured at end diastole. LV systolic function which was assessed from an apical four-chamber view by measuring the left ventricular fractional shortening (LVFS) where LV FS % = (LVEDD-LVESD)/LVEDD X 100.

Moreover, M mode echocardiography was used to measure tricuspid annular plane systolic excursion (TAPSE) in the apical four-chamber view. Special care has to be taken for the whole RV to be included in the view with no drop out in the endocardial outline along the interventricular septum and RV free wall. The width of the sector should be limited to the RV free wall, and the M-mode cursor should be positioned on the lateral portion of the tricuspid annulus, measuring in control sweep mode. Maximal tricuspid annular plane systolic excursion is defined by the total excursion of the tricuspid annulus from its highest position after atrial ascent to the peak descent during ventricular systole.

### Doppler echocardiography

Mitral valve inflow Doppler flow was measured in an apical four-chamber image, E-wave (early filling), A-wave (late filling), and the E/A ratio were derived to evaluate LV diastolic function. Pulmonary artery systolic pressure (PASP) was estimated using PASP = 4 (TR V max)^2^ + right atrial pressure.

Tissue Doppler echocardiography (TDE): the following data were assessed by TDE:

### Systolic and diastolic mitral annulus velocities

Early diastolic myocardial wave (e′), atrial diastolic myocardial wave (a′), e’/a’ ratio, systolic myocardial wave (S′), ejection time (ET), isovolumetric relaxation time (IVRT), and isovolumetric contraction time (IVCT) were measured during the baseline pulse-wave color TDI examination of the LV free wall and of the interventricular septum. The LV myocardial performance index (MPI) was calculated using the following formula: LV MPI = IVCT + IVRT/ET. Diastolic function was assessed by calculating the E/e′ ratio.

### Systolic and diastolic tricuspid annulus velocities

TDI sample volume is placed at the level of the septal tricuspid annulus. Spectral tissue Doppler (TDI) display shows an antegrade systolic wave S’, and two retrograde waves, E’ (passive LV filling) and A’ wave (atrial contraction). RV MPI was also calculated.

### 2D-STE imaging

For the STE evaluation with simultaneous ECG at frame rates of 70–100 frames/s, apical four-chamber, apical three-chamber, and apical two-chamber images were recorded in the apical view, and the parasternal short-axis view, the parasternal apical, parasternal medial, and parasternal basal images. At least four consecutive cardiac cycles were recorded for each parameter. The recorded images were transferred onto DVD and analyzed on a computer using the QLAB software (Philips Medical Systems). The mitral annulus, lateral and septal, and the LV apical endocardial planes were marked for each of the three apical chambers, allowing for the automated generation of the LV wall by the software. The LV endocardial-myocardial border was adjusted manually on the systolic frames. LV and RV global longitudinal strain were calculated automatically by the software. GLS was presented as a negative value as it measures the shortening of myocardial fibers during cardiac contraction. The more negative values indicate better myocardial function, whereas less negative values reflect impaired function.

### Statistical analysis

Statistical analysis was done by SPSS v26 (IBM Inc., Chicago, IL). Quantitative variables were presented as means and standard deviations (SD) and compared between the two groups using Student’s t-test. Qualitative variables were presented as frequency and percentage (%) and analyzed utilizing the Chi-square test or Fisher’s exact test when appropriate. Pearson correlation analysis was performed to assess the correlation between the severity of OI and other variables. A two-tailed P value is considered significant if <0.05.

## Results

Table 1 showed that the mean age of the patient group was 8.5 years, while the mean age of the control group was 9.5 years. The patient groups included 21 males and 9 females, whereas the control group included 17 males and 13 females, with no significant difference between the two groups as regards age, sex, systolic, and diastolic blood pressure. The height and weight of the patient group were significantly lower than those of the healthy control. In addition, hemoglobin and red blood cell count were significantly lower in the patient group than those of the healthy control. The mean age at first diagnosis in the patient group was 0.8 years. Regarding the severity of the disease, 12 patients (40%) had mild disease, nine patients (30%) had moderate disease, and nine patients (30%) had severe disease.

**Table 1:** Demographic, clinical, and laboratory data of the studied groups.

Parameters	Patient group	Control group	*P* value
Age (years)	8.5 ± 3.8	9.5 ± 3.4	0.399
Sex (male: female)	21: 9	17 :13	0.372
Height (cm)	111.9 ± 24.9	137.4 ± 22.3	0.002
Weight (kg)	18.2 ± 12.1	28.9 ± 6.9	0.001
Systolic blood pressure (mmHg)	93.2 ± 8.5	92.5 ± 7.8	0.165
Diastolic blood pressure (mmHg)	62.4 ± 4.7	63.1 ± 5	0.853
Red blood cells (million cells/μl)	3.8 ± 0.3	4.5 ± 0.3	0.001
Hemoglobin (g/dl)	10.8 ± 1.3	12.1 ± 1.3	0.03
Age at diagnosis (years)	0.8 ± 1.7		
Severity of the disease	- Mild 12 (40%) - Moderate 9 (30%) - Severe 9 (30%)		

Regarding echocardiographic findings of the left ventricle in the studied groups, IVS, LVPW, LVFS, mitral E/A, LV é/á, and LVMPI were comparable in both groups. AO, LA, LVESD, and LVEDD were significantly larger in the patient group than in the control group. In contrast, LV E/é, LV S, and LV 2D-GLS were significantly lower in the patient group than in the control group (Table 2).

**Table 2:** Echocardiographic findings of the left ventricle in the studied groups.

Parameters	Patient group	Control group	*P* value
AO (cm)	2.4 ± 0.3	2.1 ± 0.4	<0.001
LAD (cm)	2.7 ± 0.5	2.3 ± 0.4	<0.001
LVESD (cm)	2.4 ± 0.3	2.2 ± 0.2	0.03
LVEDD (cm)	3.8 ± 0.6	3.7 ± 0.5	0.04
IVS (cm)	0.7 ± 0.2	0.7 ± 0.1	0.231
LVPW (cm)	0.7 ± 0.1	0.6 ± 0.3	0.572
LV FS%	34.6 ± 4.8	36.4 ± 3.5	0.068
Mitral E/A	1.4 ± 0.2	1.5 ± 0.3	0.474
LV S wave (cm/s)	5.4 ± 1	6.9 ± 0.7	<0.001
LV é/á	1.5 ± 0.3	1.5 ± 0.2	0.973
LV E/é	5.3 ± 0.37	7.5 ± 2.38	<0.001
LV MPI	0.5 ± 0.14	0.4 ± 0.05	0.107
LV 2D-GLS	−17.9 ± 2.5	−21.3 ± 2.6	0.001

*AO* aorta, *LAD* left atrial diameter, *LVESD* left ventricular end systolic diameter, *LVEDD* left ventricular end diastolic diameter, *IVS* interventricular septum, *LVPW* left ventricular posterior wall, *MPI* myocardial performance index, *2D-GLS* two-dimensional global longitudinal strain.

**Fig. 1:**
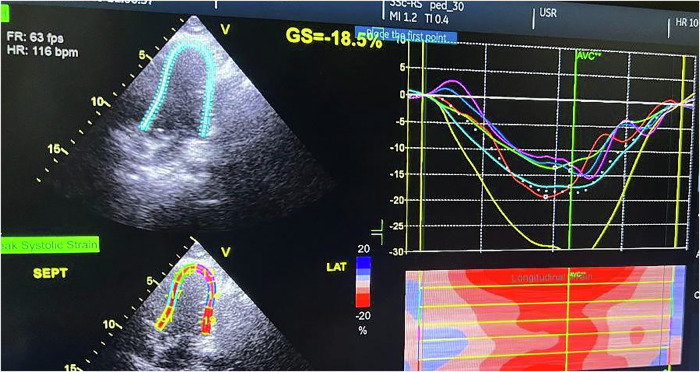
A normal LV 2D-GLS from a healthy control (LV GLS = −18.5%).

**Fig. 2:**
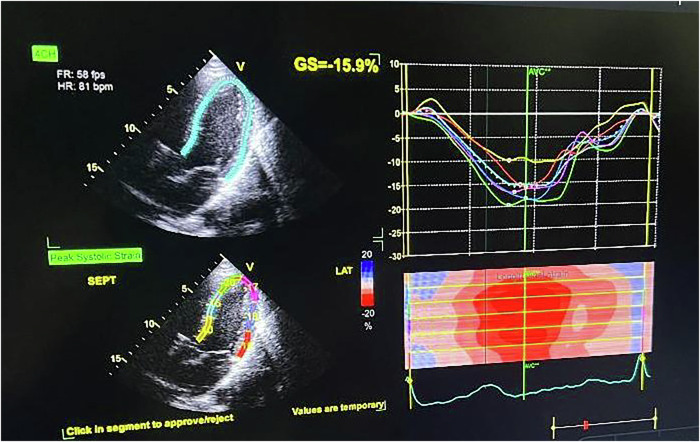
An impaired LV 2D-GLS in a patient with OI (LV GLS = −15.9%).

Figure 1 shows a normal LV 2D-GLS from a healthy control (LV GLS = −18.5%), while Fig. 2 shows an impaired LV 2D-GLS in a patient with OI (LV GLS = −15.9%).

Regarding echocardiographic findings of the right ventricle in the studied groups, TAPSE, RV S, and RV 2D-GLS were significantly lower in the patient group compared to the control group. However, RVD and MPA were significantly higher in the patient group compared to the control group. SPAP, RV é/á, and RV MPI were comparable in both groups (Table 3).

**Table 3:** Echocardiographic findings of the right ventricle in the studied groups.

Parameters	Patient group	Control group	*P* value
TAPSE (cm)	1.8 ± 0.3	2.0 ± 0.2	0.01*
MPA (cm)	2.0 ± 0.3	1.6 ± 0.2	<0.001*
RVD (cm)	1.9 ± 0.5	1.5 ± 0.3	˂0.001*
PASP (mmHg)	19.6 ± 4.5	17.8 ± 3.5	0.823
RV S (cm/s)	7.4 ± 0.7	8.5 ± 1.9	0.021*
RV é/á	1.5 ± 0.4	1.5 ± 0.2	0.543
RV MPI	0.4 ± 0.1	0.4 ± 0.1	0.324
RV 2D-GLS	−18.3 ± 4.5	24.6 ± 4.1	<0.001*

*TAPSE* tricuspid annular plane systolic excursion, *MPA* mean pulmonary artery, *RVD* right ventricular diameter, *PASP* pulmonary artery systolic pressure, *RV MPI* right ventricular myocardial performance index, *RV 2D-GLS* right ventricular two-dimensional global longitudinal strain.

**Fig. 3:**
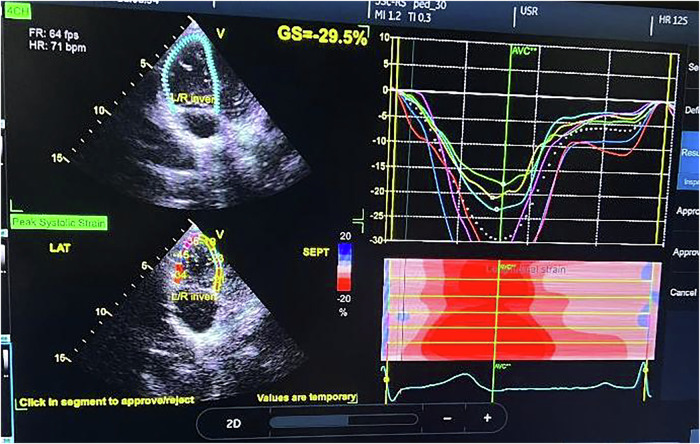
A normal RV 2D-GLS from a healthy control (RV GLS = −29.5%).

**Fig. 4:**
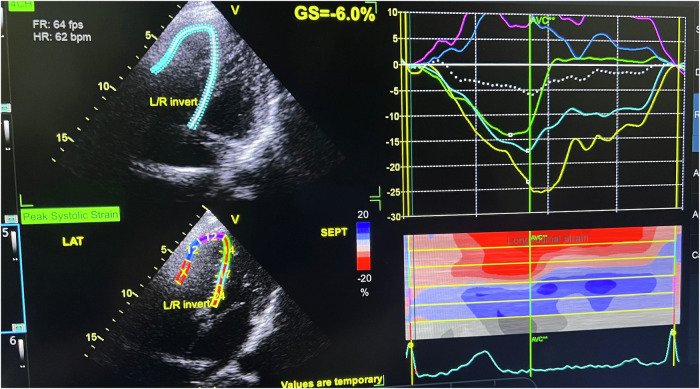
An impaired RV 2D-GLS in a patient with OI (RV GLS = −6%).

Figure 3 shows a normal RV 2D-GLS from a healthy control (RV GLS = −29.5%), while Fig. 4 shows an impaired RV 2D-GLS in a patient with OI (RV GLS = −6%).

There was no correlation between the clinical severity of the disease and (IVS, LVPW, LVFS, mitral E/A, LV S, LV é/á, LV E/é, and LV MPI). Moreover, there was a significant positive correlation between the clinical severity of the disease and (AO, LAD, LVESD, and LVEDD). However, there was a significant negative correlation between the clinical severity of the disease and LV 2D-GLS (Table 4).

**Table 4:** Correlation between echocardiographic findings of the left ventricle and severity of OI in the patient group.

Parameters	Severity of the disease	
	r	*P*
AO (cm)	0.634	0.02
LAD (cm)	0.120	0.04
LVESD (cm)	0.512	0.03
LVEDD (cm)	0.478	0.01
IVS (cm)	0.122	0.813
LVPW (cm)	0.102	0.712
LVFS%	−0.165	0.484
Mitral E/A	−0.105	0.179
LV S wave (cm/s)	0.0226	0.924
LV é/á	−0.105	0.657
E/é	−0.362	0.116
LV MPI	0.312	0.180
LV 2D-GLS	−0.516	0.03

*AO* aorta, *LAD* left atrial diameter, *LVESD* left ventricular end systolic diameter, *LVEDD* left ventricular end diastolic diameter, *IVS* interventricular septum, *LVPW* left ventricular posterior wall, *MPI* myocardial performance index, *2D-GLS* two-dimensional global longitudinal strain.

There was no correlation between the clinical severity of the disease and (TAPSE, PASP, RV S, and RV MPI). Moreover, there was a significant positive correlation between the clinical severity of the disease and both MPA and RVD. In contrast, there was a significant negative correlation between the clinical severity of the disease and both RV é/á and RV 2D-GLS (Table 5).

**Table 5:** Correlation between echocardiographic findings of the right ventricle and severity of OI in the patients group.

Parameters	Severity of the disease	
	r	*P*
TAPSE (cm)	−0.163	0.489
MPA (cm)	0.511	0.03
RVD (cm)	0.413	0.04
PASP (mmHg)	0.012	0.154
RV S (cm/s)	−0.232	0.324
RV é/á	−0.439	0.025
RV MPI	0.196	0.762
RV 2D-GLS	−0.712	0.001

*TAPSE* tricuspid annular plane systolic excursion, *MPA* mean pulmonary artery, *RVD* right ventricular diameter, *PASP* pulmonary artery systolic pressure, *RV MPI* right ventricular myocardial performance index, *RV 2D-GLS* right ventricular two-dimensional global longitudinal strain.

## Discussion

Collagen type I plays a vital role in bones and various organs, including the heart; therefore, a reduction in the amount of collagen I or changes in its biosynthesis may affect the function or structure of the heart in patients with OI.^8^ Although cardiovascular disease appears to be more prevalent in individuals with OI compared to control individuals, there are currently no clinical guidelines addressing whether children with OI should be screened for cardiac or vascular abnormalities, likely due to limited pediatric data.^9^ Hence, our study aimed to assess the cardiac function in children with OI and evaluate the need for regular cardiac follow-up in these patients.

In the present study, height and weight were significantly lower in the patient group than in the control group. This was in agreement with the results of other investigators.^10^ This may be because children with OI are poor feeders due to several factors such as limited mobility, low appetite from fractures or pain, breathing difficulties, reflux, and a weak sucking reflex. Moreover, scoliosis, lower limb deformities, and fractures of growth plates of spinal vertebrae may have an impact on the height in these children.^11^

In the current study, hemoglobin and RBC count were significantly lower in the patient group compared to the control group. This aligned with the results of Elsayed et al.^12^ Osteogenesis Imperfecta is characterized by mutations in the COLLA1 or COLLA2 genes encoding collagen type I, which plays a vital role in bone strength and function. So, the collagen defects may affect the bone marrow function, reducing the ability of the marrow to produce sufficient RBCs, thus contributing to anemia.^13^ Anemia is reported to affect cardiac function; however, most of our patients had mild anemia, and none of our patients had severe anemia, which minimized the effect of anemia on the results.

Echocardiographic findings revealed significantly larger AO, LAD, LVESD, LVEDD, RVD, and MPA in OI patients. Similarly, Zhao et al.^14^ demonstrated that children with OI had significantly larger values of LAD and LVEDD than the control group. Additionally, Pinheiro et al.^15^ reported that the OI group presented significantly larger AO, LAD, LVEDD, LVESD, and RVD compared to the control group. Furthermore, Radunovic et al.^6^ reported increased right ventricular outflow tract measurements and MPA diameters in adult OI patients, indicating involvement of both the right and left sides of the heart.

In OI, type 1 collagen fibers are altered, which is the main component of myocardial collagen and plays a major role in the stiffness and strength of the myocardial and aortic walls. These abnormalities and dilatation observed in the myocardial, pulmonary, and aortic wall echocardiographic parameters may be caused by such changes in the collagen fibers.^16^

Interestingly, conventional echocardiography failed to detect any LV systolic dysfunction. However, tissue Doppler and STE revealed LV systolic dysfunction in children with OI compared to the control group. This is due to the higher sensitivity of TDE and STE to detect subtle cardiac dysfunction before conventional echocardiography can do.^17–23^

Moreover, our study revealed that the diastolic function of the LV was impaired as revealed by significantly lower mitral E/é in the patient group compared to the control group. This agreed with the finding of Migliaccio et al.^4^ These alterations in OI patients are most likely caused by the myocardial tissue becoming more rigid and less elastic, which affects myocardial relaxation and causes diastolic echocardiographic abnormalities.

Our study also revealed impaired RV function, indicating involvement of both LV and RV. Given that over 80% of cardiac extracellular matrix is collagen type I (more than 80%) and collagen type III (more than 10%). Collagen type I fibers are particularly important for preserving the elasticity and integrity of cardiac tissue because they offer tensile strength and resistance to deformation. Furthermore, they are essential for maximizing the heart’s pumping efficiency by transferring mechanical forces from the extracellular matrix to the cardiomyocytes and vice versa. An imbalance in collagen metabolism causes structural and functional issues with the heart, such as cardiac fibrosis or following myocardial infarction. The myocardial structure and function may therefore be impacted by abnormal or reduced collagen type I defects.^16^

Our study revealed that there was a significantly negative correlation between the clinical severity of disease and RV é/á, RV 2D-GLS, and LV 2D-GLS. This was expected as the more severe the disease, the more the systolic and diastolic dysfunction of the heart.

Our study supports the hypothesis that patients with OI may undergo mild alterations in their cardiovascular anatomy and function, and that the severity of the disease may affect the degree of these alterations. Moreover, our study highlights the need for early, detailed assessments of cardiac function in children with OI. Although our research demonstrated that most echocardiographic abnormalities are mild, asymptomatic, and without the need for medical intervention, we recommend that children with OI should have baseline echocardiography as early as possible and frequent follow-up for the progression of cardiac lesions.

### Limitations of the study

Being a single-center study, relatively small sample size, a lack of follow-up of the patients by echocardiography, and the absence of 3D-STE from our echocardiography software were the main limitations of the study.

## Conclusion

Children with OI have subclinical LV and RV systolic and diastolic dysfunction, and these abnormalities were correlated with the clinical severity of the disease. In addition, they have larger AO, LA, LVESD, LVEDD, RVD, and MPA than the control group. Regular echocardiographic follow-up is recommended in these patients.

## Data Availability

All the data of the study are available from the corresponding author on reasonable request.
